# Intracellular Zinc Trafficking during *Crotalus atrox* Venom Wound Development

**DOI:** 10.3390/ijms24076763

**Published:** 2023-04-05

**Authors:** Eric A. Albrecht, Jasmine D. Carter, Veronica Garbar, Abeeha Choudhary, Scott A. Tomlins

**Affiliations:** 1 Department of Molecular and Cellular Biology, Kennesaw State University, Kennesaw, GA 30144, USA; 2 Department of Pathology, Michigan Medicine, University of Michigan, Ann Arbor, MI 48109, USA

**Keywords:** *Crotalus atrox*, wound, regeneration, zinc, cytotoxicity, venom, injury

## Abstract

In this study, we examined zinc trafficking in human umbilical vein endothelial cells (HUVEC) stimulated with *Crotalus atrox* (CA venom) snake venom. We utilized MTS cytotoxicity assays to monitor the cytotoxic range of CA venom. HUVEC monolayers stimulated with 10 µg/mL CA venom for 3 h displayed cellular retraction, which coincided with 53.0 ± 6.5 percent viability. In contrast, venom concentrations of 100 µg/mL produced a complete disruption of cellular adherence and viability decreased to 36.6 ± 1.0. The zinc probe Fluozin-3AM was used to detect intracellular zinc in non-stimulated controls, HUVEC stimulated with 10 µg/mL CA venom or HUVEC preincubated with TPEN for 2 h then stimulated with 10 µg/mL CA venom. Fluorescent intensity analysis returned values of 1434.3 ± 197.4 for CA venom demonstrating an increase of about two orders of magnitude in labile zinc compared to non-stimulated controls. Endothelial response to CA venom induced a 96.1 ± 3.0- and 4.4 ± 0.41-fold increase in metallothionein 1X (MT1X) and metallothionein 2A (MT2A) gene expression. Zinc chelation during CA venom stimulation significantly increased cell viability, suggesting that the maintenance of zinc homeostasis during envenomation injury improves cell survival.

## 1. Introduction

Wound formation involves the disruption of the structural elements of tissues (e.g., various cell types, structural proteins). Destabilization of tissue structure triggers platelet exposure to collagen and extracellular matrix followed by vasoconstriction, coagulation, and acute inflammatory signaling. Current understanding of the wound and regeneration process has focused on cellular responses to wound events. For example, the migration of fibroblast, macrophage, and neutrophils to the site of injury and the complex signaling events that occur to mitigate the injury. However, more evidence suggests that injured tissues contribute to the early stages (hours) of wound development and wound healing [1,2]. Studies examining the effects of envenomation can provide insight into the response of tissues to noxious stimuli at early time points (≤3 h).

Edema and internal hemorrhaging describe clinical manifestations of CA venom envenomation. At the envenomation site, venom reduces cellular connections to the extracellular matrix, triggering apoptosis and local tissue necrosis. This type of complex wound is often difficult to heal, and, if allowed to progress, it can create permanent disfigurement or death. Proteolytic proteins in CA venom such as snake venom metalloproteases and serine proteases exert their activity on tissues at the site of envenomation and delay wound regeneration. Snake venom metalloproteinases (SVMP) comprise a large percentage of the secreted proteins in CA venom and are known to cause hemorrhaging [3]. This activity stems from SVMP’s ability to inhibit platelet aggregation and blood serine proteases inhibitors in combination with activating prothrombin among other blood proteins. SVMP found in CA venom (e.g., atroxase, atrolysin A-D, vascular apoptotic protease 1 and vascular apoptotic protease 2) are known to hydrolyze collagen, fibrin, and fibrinogen [4,5,6,7]. More recent studies suggest disintegrin domains in SVMP, such as catrocollastatin C, may be involved in collagen inhibition and contribute to degradation of vascular integrity [8,9]. Thus, SVMP contribute to local pathology by decreasing tissue integrity. Additional proteolytic actions are carried out by serine proteases. 

CA venom contains serine proteases such as catroxase-1, catroxase 2, Kal-likrein-like EII and α-fibrinogenases which degrade components of the blood coagulation components cascade or selectively inhibit blood factors involved in clotting and fibrinolysis [10]. The fibrin (ogen)olytic activity of serine proteases toward the Bb-chain of fibrinogen contributes to tissue destruction at envenomation sites. Proteolytic destruction of tissue by SVMP and serine proteases work in parallel with non-enzymatic proteins such as disintegrins, C-type lectins and C-type lectin-like proteins to increase cellular substrate detachment.

Disintegrins are non-enzymatic, cysteine-rich proteins shown to originate from proteolytically processed SVMP precursors; thus, they possess structural domains similar to SVMP. Disintegrins structurally fall into monomer, homodimer or heterodimer classifications and contain R/KTS, RGD, or MLD motifs which bind to integrins and competitively inhibit substrate attachment [10]. Several disintegrins (e.g., atroxatin and crotatroxin) found in CA venom promote loss of cellular adherence to the extracellular matrix [11,12]. For example, crotatroxin reportedly inhibits β1α5, β3αv and αIIb binding to fibronectin, collagens, VWF and other structural proteins [13]. Similarly, C type lectins and C-type lectin-like proteins in CA venom reduce tissue integrity. C type lectins in CA venom (e.g., rattlesnake lectin (RSL)) contain calcium and sugar binding capacity, such as selectins and mannose binding proteins, which act to induce erythrocyte agglutination. C-type lectin-like proteins may inhibit or activate platelets; however, C type lectin-like proteins do not contain the classic calcium/sugar-binding loop found in C type lectins [14]. Two C Type lectin-like proteins from *Calloselasma rhodostoma* (e.g., Rhodocetin and Aggretin) bind the collagen receptor α_2_β_1_, suggesting C type lectin-like components can contribute to the loss of cellular adherence during envenomation by inhibiting collagen–integrin binding [15]. Other identified venom components such as myotoxins and L-amino oxidase likely contribute to pro-apoptotic complications during venom-induced tissue injury. L-amino oxidases (e.g., Apoxin I) induce H_2_O_2_ formation by the oxidation of L-amino acids [16], and affect the execution of apoptotic programming by altering intracellular redox environments. Increasing evidence suggests they contribute to pro-inflammatory and apoptotic signaling cascades in a variety of cell types. 

The combined activity of SVMP, serine proteases, disintegrins, C-type lectin, and other proteases found in CA venom initiate loss of adherence to the extracellular matrix. Decreased cellular adherence reduces integrin survival signaling and activates several intrinsic pro-apoptotic signaling cascades that degrade mitochondrial integrity by the activation of the pro-apoptotic proteins, such as Bad and Bax [17,18]. During loss of adherence (anoikis), the anti-apoptotic protein Bcl-2 and Bcl-xl activation counteracts mitochondrial injury by inhibiting pore formation in the outer membrane [19,20]. In parallel, Akt survival pathways promote phosphorylation of mTOR, the sequestration of Bad from the mitochondria, and NFkB-mediated transcription of anti-apoptotic genes [21,22].

Other anti-apoptotic mechanisms include intracellular zinc binding to pro-apoptotic proteases. Initial studies examining zinc’s role in apoptosis demonstrated that zinc can lower the activity of caspase-3 [23,24]. In contrast, investigations using epithelial cells have reported that excessive intracellular zinc concentration induces apoptosis [25]. Thus, zinc-mediated apoptosis regulation may be concentration-dependent, but the extent to which this contributes to venom-induced tissue pathology is unclear. Within cells, 20–30% of the zinc is bound to proteins in the nucleus, and the remainder is located in the mitochondria, endoplasmic reticulum and/or metallothioneins in the cytosol. Metallothioneins represent the main cytosolic zinc-binding proteins and are classified as early stress responders [26,27]. 

Metallothionein gene induction involves the binding of labile intracellular zinc to the cytosolic transcription factor, metal transcription factor-1 (MTF1). Translocation of MTF1 and its binding to DNA metal response elements (MRE) initiate metallothionein transcription. In an earlier study, our group reported that *Echis carinatus* venom induced a significant up-regulation of metallothionein genes in HUVEC after 3 h stimulation [28], suggesting zinc mobilization is a part of the injury response. It is unclear if increased intracellular zinc promotes cell survival, because recent studies indicate that mobilization of intracellular zinc can trigger mitochondrial dysfunction, increase oxidative stress, and cell death [29,30,31]. In addition, labile Zn pools might interfere with normal functioning of mitochondria and contribute to apoptosis [32]. Several groups have examined the use of Zn^2+^ chelators as potential venom therapies; however, excessive zinc depletion induces apoptosis in several cell types [33,34]. This therapy has been investigated as potential way to control SVMP activity, since several of the SVMP have zinc cofactors. Here, we describe early cellular injury events and investigate intracellular zinc trafficking as a part of wound development. 

## 2. Results

### 2.1. CA Venom Decreases Cell Viability and Apoptotic Related Proteins

**Figure 1:**
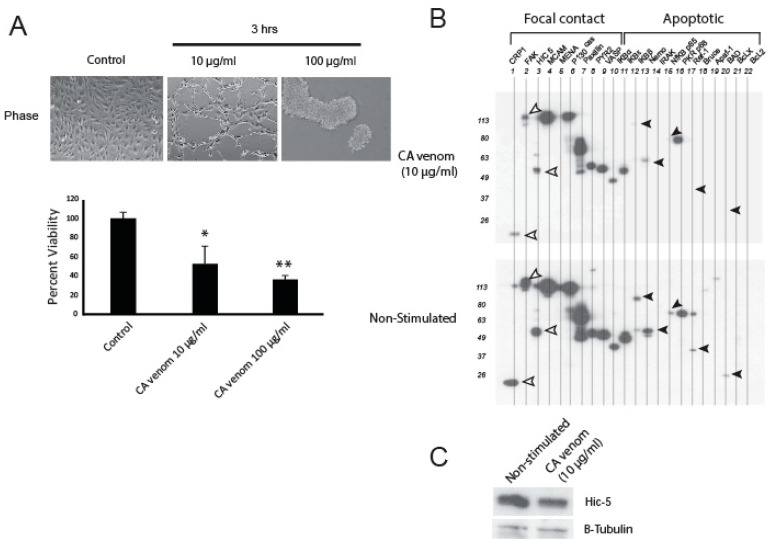
CA venom induces loss of substrate contact and decreased viability in HUVEC. (**A**) Phase contrast images of HUVEC monolayers stimulated with 10 or 100 µg/ml CA venom for 3 h. MTS assay demonstrates significant decrease in HUVEC viability after 3 h stimulation with 10 or 100 µg/ml CA venom. * *p* < 0.05 vs. control ** *p* < 0.01 vs. non-stimulated control (n = 3). (**B**) Multi-Western blotting of focal contact and apoptotic proteins. HUVEC monolayers stimulated with CA venom (10 µg/mL) for 3 h. Focal contact proteins indicated by white arrow and apoptotic proteins (black arrows). Control included non-stimulated lysates. (**C**) Conventional Western blotting of Hic-5 and β-tubulin loading control.

In an earlier report, we demonstrated that hemorrhagic venom induces loss of adherence to the extracellular matrix and cytotoxicity in HUVEC [29]. The early events (≤3 h) associated with CA venom stimulation were examined to determine the sublethal concentrations of the venom that would allow measurement of intracellular zinc via microscopy. We monitored cell morphology and tested the cytotoxic range of CA venom after 3 h stimulation (Figure 1A). Phase contrast images of HUVEC monolayers stimulated with 10 µg/mL CA venom displayed cellular retraction, which coincided with 53.0 ± 6.5 percent viability, in contrast to non-stimulated control monolayers which had little or no retraction and near 100 percent viability. At a venom concentration of 10 µg/mL, the cells showed mild retraction from the substrate, generating punctuated clear surface areas within the monolayers. Similarly, HUVEC stimulated for 3 h with venom concentrations of 100 µg/mL showed 36.6 ± 1.0 percent viability (Figure 1A) and cell adherence was completely abolished. In addition, we examined the qualitative expression of several focal contact and apoptotic-related proteins in response to venom treatment (Figure 1B). To do this, we employed a multi-blot apparatus that allows for simultaneous detection of proteins that are detected by primary antibodies raised in the same animal. Lysates were collected from cells stimulated for 3 h with CA venom (Figure 1B, upper blot) and non-stimulated cells (Figure 1B, lower blot). Multi-Western blotting results reveal that stimulated cells have decreased expression in focal contact proteins Cysteine-rich protein-1 (CRP-1), Focal Adhesion Kinase (FAK) and Hic-5 (Figure 1B, white arrows). We observed similar decreases in expression for apoptotic signaling proteins (black arrows) such as IKBε, IKBβ, Nfκβ 65p, redox factor 1 (Ref-1) and a BH3-only pro-apoptotic protein (BAD) after stimulation with CA venom for 3 h. The lysates were probed for Hic-5 using conventional SDS-PAGE and Western blotting techniques to confirm expression pattern of the multi- Western blot (Figure 1C).

### 2.2. CA Venom Cellular Injury Induces Increases in Intracellular Labile Zinc

**Figure 2:**
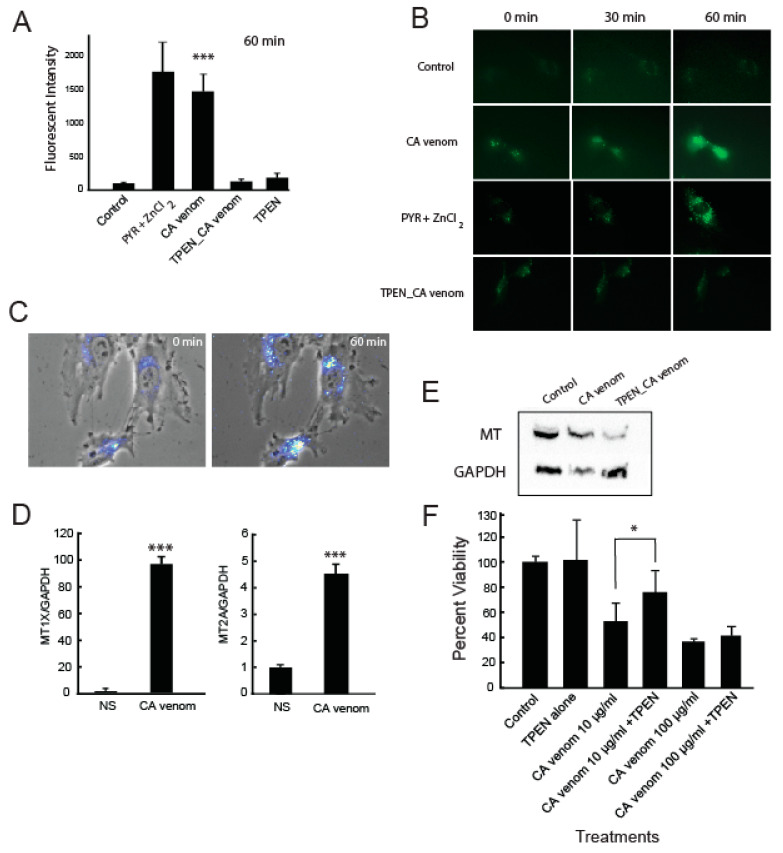
Zinc trafficking in HUVEC during CA venom stimulation. (**A**) Fluorescent microscopy analysis. Cells were stimulated with CA venom, pyrithione-zinc, TPEN_CA venom or TPEN alone for 1 h. *** *p* < 0.001 vs. non-stimulated control (n = 10–15 cells per experimental group). (**B**) Representative fluorescent micrograph time course of HUVEC loaded with FluoZin-3AM treated with PYR/ZnCl_2_, TPEN (preincubation) then CA venom or CA venom alone (400×). (**C**) Phase contrast and fluozin-3AM fluorescent composite micrographs of HUVEC stimulated with CA venom after 60 min period. (**D**) qPCR of MT1X and MT2A gene expression after 3 h stimulation with CA venom *** *p* < 0.001 (n = 3). (**E**) Western blot. Metallothionein (MT) protein expression stimulated with CA venom or TPEN (preincubated) then CA venom for 8 h. (**F**) MTS cytotoxicity assay. Cell treated with CA venom alone (10 and 100 μg/mL), TPEN alone 3 µM or pretreated with 3 μM TPEN, then stimulated with CA venom (10 or 100 μg/mL) or 3 h. * *p* < 0.05 vs. CA venom 10 μg/mL.

To determine whether intracellular zinc mobilization occurs as an immediate early response to CA venom stimulation, we used the cell permeant fluorescent zinc probe Fluozin-3AM which binds to intracellular labile zinc. Non-stimulated control cells emit low levels of fluorescence, corresponding to the binding of Fluozin-3AM to labile zinc pools or parts of the cellular proteome coordinated with zinc [35,36]. During stimulation, changes in intracellular labile zinc can be measured by changes in baseline fluorescent intensity (Figure 2A). HUVEC stimulated with 10 µg/mL CA venom for 1 h displayed an average fluorescent intensity of 1434.3 ± 197.4, which was approximately two orders of magnitude greater than that of non-stimulated controls (*p* ≤ 0.001). The zinc ionophore PYR was used as a positive control of labile zinc and returned fluorescent intensity values of 1605.9 ± 273.6, closely matching the values reported for CA venom-stimulated cells. Cells pre-incubated with the zinc chelator TPEN then stimulated with 10 µg/mL CA venom displayed an average fluorescent intensity of 128.1 ± 34.9. 

To better understand the spatial distribution of zinc prior to and during CA venom stimulation, we imaged cells using a 40× semi-apochromat objective (Figure 2B). Figure 2B demonstrates the intracellular distribution and changes in FluoZin3AM fluorescence during non-stimulating and CA venom stimulation conditions. Non-stimulated cells displayed punctuated fluorescence matching a previous report [37]. During CA venom stimulation, the pattern of intracellular fluorescence became more diffusely distributed (Figure 2B). Figure 2C represents composite images of HUVEC stimulated with CA venom for 1 h. Fluozin3AM fluorescence is perinuclear and develops in intensity over time.

Next, we monitored the gene expression of metallothionein 1X (MT1X) and metallothionein 2A (MT2A) during CA venom stimulation to determine whether changes in their expression coincided with increases in intracellular zinc (Figure 2D). The gene expression of MT1X and MT2A was monitored by quantitative PCR (qPCR) after 3 h stimulation with 10 µg/mL CA venom. Results showed the gene expression of MT1X and MT2A increased by 96.1 ± 3.0- and 4.4 ± 0.41-fold, respectively. In addition, the proteomic changes to metallothioneins were evaluated using an antibody that detects both MT1X and MT2A isoforms (MT) (Figure 2E). Results showed a moderate increase in metallothionein expression over an 8 h stimulation period. The TPEN_CA venom experimental group showed a significant reduction in metallothionein protein expression compared to non-stimulated and venom alone-stimulated groups, suggesting zinc trafficking occurs during CA venom stimulation.

To determine whether increases in intracellular zinc negatively or positively impacted cell survival, we used the cell permanent zinc chelator TPEN to lower intracellular labile zinc released during venom stimulation. We monitored cell viability by MTS assay under the identical stimulating conditions. Figure 2F shows 10 ug/mL CA venom induced 52.9 ± 6.5% reduction in cell viability after 3 h, supporting the same pattern shown in Figure 1A. In contrast, pre-incubation of cells with 3 µM TPEN significantly improved cell viability by 23.1% for cells stimulated with 10 µg/mL CA venom. 

## 3. Discussion 

Envenomation by *Crotalus atrox* produces significant tissue damage; however, the intracellular signaling mechanism(s) that characterize envenomation injuries are not fully resolved. Local envenomation injury becomes visible within 3 hours. Therefore, we chose this time point to evaluate cellular responses to sublethal concentrations of CA venom. During our evaluation of CA venom injury formation, we determined that cell viability and adherence to the extracellular matrix was concentration-dependent (Figure 1). This is well reported in other studies, suggesting that the loss of adherence may be an important factor to envenomation injuries [38,39]. For example, Pierce et al. reported that HEK293 cells stimulated with *Echis carinatus* venom for 3 h did not show a loss in membrane integrity and that maintaining attachment to the extracellular matrix was directly proportional to cell survival [39]. In this study, cell viability was measured using an MTS assay, which monitors mitochondrial metabolic rates and suggests venom stimulation initiates intrinsic apoptotic pathways. This was further supported with immunoblotting results demonstrating a decrease in pro- and anti-apoptotic proteins. Figure 1B shows an early reduction in IκBβ and APE/Redox factor 1 (Ref-1). Ref-1 mediates DNA binding to a number of transcription factors, including NfκB [40,41]. NfκB is a multimeric protein that exerts transcriptional control of cell survival proteins [42,43]. Thus, immediate early responses (≤3 h) to CA venom stimulation include reduction in NfκB-mediated cell survival signaling. Although, beyond the scope of this communication, the loss of NfκB signaling proteins may be connected to excessive labile intracellular zinc. Using TPEN to chelate the zinc and monitor the expression of NfκB signaling proteins may provide insight into other aspects of injury induce zinc trafficking.

In an earlier report, we determined that stimulating HUVEC for 3 hr with hemorrhagic venom (*Echis carinatus*) induced the up-regulation of metallothionein genes [28]. The up-regulation of metallothionein genes indirectly suggested zinc trafficking may be a part of that injury response. In this study, we monitored intracellular zinc mobilization after one hour of CA venom stimulation using the fluorescent probe FluoZin 3AM. Significant increases in labile zinc (Figure 2) occurred in response to CA venom. The zinc ionophore PYR was used as a positive control and induced a rapid increase in intracellular zinc [29]. Importantly, we determined that significant increases in intracellular labile zinc occur within 1 hour of venom stimulation and that this increase approaches intracellular levels recorded for our positive control PYR. Metal toxicity involves disruption of disulfide bonds or displacement of metal cofactors in metabolic enzymes. Although zinc is an important bio-metal for cellular functions, excessive concentrations of zinc introduced by PYR and/or CA venom may competitively inhibit key metabolic processes [29]. We hypothesized that venom-induced intracellular increases in zinc may trigger downstream zinc-mediated signaling involving metal transcription factor 1 (MTF1), which is known to activate metallothionein gene transcription. Therefore, we examined the gene activation of MT1X and MT2A after 3 hours of CA venom stimulation. Our results demonstrated a dramatic increase in MT1X (96.1-fold) and MT2A (4.4-fold) gene expression. Pre-incubation of HUVEC with TPEN prior to CA venom stimulation resulted in a reduction in MT protein expression, providing additional support for the occurrence of zinc trafficking during CA venom stimulation. 

High intracellular levels of zinc are known to be damaging and pro-apoptotic in many cell types [44,45,46]. Several signaling pathways activated in response to venom are linked to alterations in intracellular zinc. For example, Qitao et al. demonstrated that crude rattlesnake venom utilizes a β4 integrin-mediated signaling pathway, leading to the up-regulation of p53 gene expression [47]. p53 is a stress-activated transcription factor, shown to be positively regulated by intracellular zinc [48]. An additional study by Qitao et al. showed that rattlesnake venom induces apoptosis through phosphatidylcholine phospholipase (PC-PLC) and this involves increased protein expression of p53 [49]. Zinc is necessary for PC-PLC enzymatic activity, and PLC inhibition by the inhibitor D609 is mechanistically achieved through the chelation of zinc from the active site [49]. This suggests rising intracellular zinc during CA venom stimulation may increase p53 transcriptional activity of pro-apoptotic protein such as BAX-1 and PIG-3. Although we did not specifically examine PC-PLC, the addition of TPEN to HUVEC prior to venom stimulation may have suppressed PLC signaling pathways resulting in increased cell viability. Thus, initial loss of zinc homeostasis may contribute to decreased cell viability during venom stimulation. In this report, we focused on immediate early events associated with cellular venom injury. However, extending the stimulation periods and examining the wound regeneration process may provide additional insight about wound care.

Several reports showed that the use of chelators successfully controls the hemorrhagic activity of venom [50,51]. We used TPEN to control intracellular zinc levels during CA venom stimulation. The results suggested this improved cell viability. The beneficial reduction in SVMP activity, obtained by zinc chelation, may also improve the cellular response to tissue destruction imposed by the venom. Since CA venom contains several proteins that induce loss of cellular adherence, independent of zinc’s presence (e.g., serine protease, disintegrins and SVMP) the therapeutic value of controlling intracellular zinc levels in tissue during envenomation deserves consideration. 

## 4. Material and Methods

### 4.1. Endothelial Cell Culture and Venom Preparation

Pooled HUVEC were cultured in basal media (LifeLine Cell Technology, Frederick, MD, USA) supplemented with growth factors and 10% fetal calf serum. Cells were grown in an incubator that maintained 5% CO_2_ and 37 °C. All experiments were performed using the first 3–4 cell passages. Lyophilized CA venom (Sigma-Aldrich, St. Louis, MO, USA) was rehydrated in phosphate buffered saline (PBS), diluted to 1 mg/mL, aliquoted, and then stored at −80 °C. A new aliquot was used for each assay. 

### 4.2. MTS Assay

HUVEC cell suspensions were plated into 96-well plates coated with 1% gelatin, seeded at 1 × 10^5^ cells/mL and allowed to attach for 18 h. Confluent, HUVEC were stimulated with 10 µg/mL or 100 µg/mL CA venom for 3 h. Additional stimulating conditions included pre-incubation (2 h) and removal of 3 µM N,N,N,N-Tetrakis (2-pyridylmethyl)-ethylenediamine)(TPEN) (Sigma, St. Louis), followed by stimulation with 10 µg/mL CA venom for 3 h. Controls included non-stimulated cells and cells in the presence of TPEN alone. After 1.5 h, 20 µL of CellTiter 96^®^ Aqueous One Solution Reagent (Promega, Madison, WI, USA) was added to each well, incubated at 37 °C for 1.5 h, then read at 490 nm. In addition, changes in cell morphology were observed using an Olympus IX71 microscope coupled to an Olympus DP71 charged coupled device (CCD) camera. Phase contrast images were captured using long working distance DIC/Phase condenser with 0.55 numerical aperture fitted with 10× and 20× phase contrast annulus rings. 

### 4.3. Quantitative PCR

Gene expression was monitored using real-time reverse transcriptase polymerase chain reactions. Briefly, HUVEC cultures were stimulated for 3 h with 10 µg/mL CA venom in triplicate. The total RNA from each replicate was isolated, then pooled. One microgram of total RNA was reverse-transcribed, producing cDNA, and stored at −20 °C. Quantitative PCR was performed using Applied Biosystems Prism 7000 Sequence Detection System in combination with an SYBR green PCR Master Mix (Applied Biosystems, Foster city, CA, USA). Reactions were assembled using 1 ng of cDNA, gene-specific primers (IDT Inc., Coralville, IA, USA) in 96-well optical grade PCR plates. GAPDH gene was used as an internal control as previously described [28]. 

### 4.4. Western Blotting

For multi-blotting, HUVEC were grown to confluence, then stimulated with CA venom (10 µg/mL) alone for 3 h. Negative controls included non-stimulated cell lysates. Additional experimental stimulating conditions included pre-incubation with 3 µM TPEN for 2 h, then stimulation with 10 µg/mL CA venom for 8 h. After stimulation, the media were removed, and the monolayer was washed twice with 37 °C PBS. Next, the PBS was removed, lysing buffer added (50 mM Tris-HCl pH 8.0, 1% NP40) containing protease inhibitors (Roche, Mannheim, Germany) and then placed on ice. Monolayers were disrupted by cell scrapers, sonicated and centrifuged at 13,500 rpm for 5 min. BioRad DC protein colorimetric assay was used to determine total protein concentrations. Standardized supernatants were combined with sample buffer containing 2-betamercaptol ethanol and stored at −80 °C. The cell lysates were subjected to 10% sodium dodecyl sulfate poly acrylamide gel electrophoresis (SDS-PAGE) and transferred to 0.2 µm nitrocellulose membranes. Following transfer, membranes were blocked with 5% non-fat dry milk dissolved in TBST (1 M Tris pH 8, 1 M NaCl, 0.1% Tween 20) for 1 h. 

For multi-blots, the blots were secured in a multi-blot apparatus (BioRad, Hercules, CA, USA). Focal and apoptotic proteins were immunoblotted using a 1:2000 dilution of anti-mouse antibodies (BD Biosciences, San Jose, CA, USA). Conventional single target blots were incubated with anti-mouse antibodies to Hic-5 (BD Bioscience, 1:2000 dilution) or anti-mouse metallothioneins (GeneTex, 1:1000 dilution). Both methods included an overnight incubation at 4 °C and then washing with TBST twice. Matching secondary antibody coupled to horseradish peroxidase in 5% milk were incubated with the blots for 2 h at room temperature, then washed twice with TBST and TBS. The immunoreactive bands were detected using enhanced chemiluminescence (ECL) (Pierce), and visualized with Chemi-Doc XS systems (BioRad, Hercules, CA, USA). 

### 4.5. Fluorescent Intensity Assay and Microscopy Imaging

HUVEC cells were plated into sterile chambered cover-glass containers (Fisher Scientific, Waltham, MA, USA) and incubated at 37° with 5% CO_2_ overnight. Following incubation, cells were incubated with 3 µM zinc pyrithione (PYR), 10 µg/mL CA venom, 10 µg/mL CA venom pre-incubated with 3 µM TPEN (TPEN_CA venom) for 2 h. For HUVEC monolayers pre-incubated with the zinc specific chelator TPEN (3 µM), media were removed, the monolayers were washed 2X with PBS and then stimulated with 10 µg/mL CA venom. Microscopy was performed by using an Olympus IX71 coupled to a charge-coupled device camera (DP71, Olympus) and stored on a Dell Omi-Plex. Fluorescent images were recorded using FITC (HQ480 (EX)/Q505LP(BS)/HQ535(EM) filter sets and a 20× objective. Tiff images were loaded into Image J [52] and intensity values recorded of randomly selected cells. All recorded raw sample intensity values where background subtracted, then normalized by captured area and the average fluorescent intensity of matched controls. High magnification images (400×) where captured using a UPlanFLN40XPh objective. Captured images were processed using image J.

### 4.6. Statistical Analysis

Statistical analysis of MTS and FluoZin 3AM assays was carried out in Excel using the add-in XRealStats or Prism 3.0 Software. Cell viability (MTS) was calculated as percent of the non-stimulated control, graphed as mean ± STD, and represented by three biological replicates. FluoZin 3AM assays included matched non-stimulated controls and consisted of 10–15 cells per treatment. Data were evaluated using Shapiro–Wilk test for normality and further analyzed by two-sided student *t* tests. Data represented by STD.

## Data Availability

The data presented in this study are available on request from the corresponding author.
